# St. Thomas's Hospital

**Published:** 1829-10-01

**Authors:** 


### XIII.

### SAINT THOMAS'S HOSPITAL.

[Mr. BURY, Reporter.]

#### I. VENEREAL SORE ON THE MAMMA, FROM SECONDARY SYPHILIS IN THE MOUTH OF A CHILD AT THE BREAST.

A woman, æt. 40, applied at St. Thomas's Hospital January 9th, 1829, having an elevated sore, of a suspicious character, nearly the size of a shilling, on the left breast, to the right of nipple. Its surface was slightly excavated in the centre; smooth, having no observable granulations; and the discharge from it was small, thin, and bloody—there were great pain and tenderness, and some hardness of its base. One of the axillary glands was enlarged to the size of a chesnut. This, the patient stated, had been the case for nearly six weeks, at which distance of time she also said she first perceived the sore on the breast. The latter, from her account of its nature when it first appeared, and from the description she gave of its subsequent mode of increase, which were exactly those of a primary syphilitic ulcer, was believed to be such, and even its appearance alone would have guaranteed such an opinion.

The patient attributed the origin of the ulcer to giving suck, for some weeks before its first appearance, to the child of another woman, who was known to have had the venereal disease—whether at the time the infant was born, or whether it had been cured in her before its birth, we could not distinctly ascertain, though it would have been satisfactory to have known; but we should suspect the former. The child, about six weeks after birth, had ulcers of the mouth and blotches on the trunk, and when in this condition, it was applied to the patient's breast.

The aspect of the sore being so decidedly specific, Mr. Green thought the present a favourable opportunity to ascertain whether a sore of this kind, viz. one primarily syphilitic, could be produced by the local application of the matter of ulcers belonging to the secondary form of lues. This, he considered, might be brought to the test of proof by waiting the result of the case, without the interposition of the specific remedy. Some arg. nitr. was applied to the ulcer, and aperient medicine, with pulv. ipec. co. given internally.

Feb. 5th. The woman came to-day, having iritis of the left eye, which came on about a week ago. The sore on the mamma somewhat resembled a wart, there being no raw surface, and, consequently, no secretion from it. Its surface was smooth, as before, and elevated abruptly—the hardened base remained. Caustic had been applied to it twice or three times. The ring round the margin of the cornea was very distinct. The iris itself was but little altered in colour, and exhibited no deposit. Calomel, gr. ij. Opii, gr. ss. M. 2dis horis.

6th. Pain and redness of conjunctiva having increased, leeches were applied.

9th. Gums and mouth sore; iris restored to colour and moveable; vascularity of conjunctiva much less. Has not taken the medicine since the night before last, when she first felt her mouth becoming sore; she then left it off of her own accord. The sore of the mamma not so elevated, but its surface appears raw. Ordered to resume the calomel and opium.

A few days after this period we again saw the woman. Very slight remains of the iritis, as well as of the sore on the breast, were then apparent. All the particulars of the case being considered, together with the occurrence of iritis, little doubt was now entertained of the specific nature of the sore

This, then, we believe to be an instance, wherein a primary syphilitic ulcer had its origin from the matter of secondary lues coming in contact with a sound and healthy skin—it being in a situation not obnoxious to that of a primary ulcer or chancre.

II. INFLAMMATION OF CELLULAR COAT OF VENA SAPHENA MAJOR.

John Anderson, æt. 11, admitted January 24, 1829, under Mr. Travers. He has an irritable sore, in chief superficial, at the base of the fifth toe, on the dorsal surface. This has existed for a fortnight, being produced by wearing a tight shoe, and has been much aggravated by daily exercise. About the middle of metatarsus, an inflamed line, an inch broad, commences, and extends upwards to the groin, along the course of the saphena major. The redness is shining, transparent, and indicates the inflammation to be of a passive kind; it is more conspicuous, for a short space, in the middle of the leg; it then becomes less so for a few inches above and below the knee, but is very distinct in the thigh. The lower tier of inguinal glands slightly enlarged and tender. The inflamed line is hardened, and has a knotted feel. The boy's aspect is unhealthy, and evinces debility, otherwise his constitution is surprisingly little disturbed. He describes the pain as severe, but we do not think this arises entirely from the action going on in the inflamed parts. He has experienced no chills, and has slept well at night. He was placed immediately in bed, with the limb supported on pillows. Twenty leeches to be applied along the part, and a dose of jalap and calomel.

25th. Has passed a tranquil night—redness and tenderness in the course of the vein considerably diminished. Bowels open. Rep. hirudines.

27th. The redness remains only over the tibia—the parts are not softer, but in the groin and throughout the entire line, formerly so well marked, the tenderness is sensibly lessened. The lobulated or knotted feel to the fingers is not so perceptible.

31st. Inflammation has progressively subsided—there is some tenderness still, and likewise induration in the course of the vein and at the groin.

Feb. 7th. Convalescent—hardness not removed.

11th. Presented; the affected part being a little harder than natural.

In Mr. Travers' opinion, the *external* coat of the vein was alone implicated, though, for obvious reasons, such could not be very distinct. In this case, there was no œdema similar to that occurring in phlegmatia dolens.

